# Detecting Parkinson’s disease and its cognitive phenotypes via automated semantic analyses of action stories

**DOI:** 10.1038/s41531-022-00422-8

**Published:** 2022-11-25

**Authors:** Adolfo M. García, Daniel Escobar-Grisales, Juan Camilo Vásquez Correa, Yamile Bocanegra, Leonardo Moreno, Jairo Carmona, Juan Rafael Orozco-Arroyave

**Affiliations:** 1 Global Brain Health Institute, University of California, San Francisco, USA; 2 Cognitive Neuroscience Center, Universidad de San Andrés, Buenos Aires, Argentina; 3 National Scientific and Technical Research Council (CONICET), Buenos Aires, Argentina; 4 Departamento de Lingüística y Literatura, Facultad de Humanidades, Universidad de Santiago de Chile, Santiago, Chile; 5 Latin American Brain Health Institute (BrainLat), Universidad Adolfo Ibáñez, Santiago, Chile; 6 GITA Lab, Faculty of Engineering, Universidad de Antioquia UdeA, Medellín, Colombia; 7 Fundación Vicomtech, Basque Research and Technology Alliance (BRTA), Donostia, San Sebastián Spain; 8 Grupo de Neurociencias de Antioquia, Facultad de Medicina, Universidad de Antioquia, Medellín, Colombia; 9 Grupo Neuropsicología y Conducta (GRUNECO), Facultad de Medicina, Universidad de Antioquia, Medellín, Colombia; 10 Sección de Neurología, Hospital Pablo Tobón Uribe, Medellín, Colombia; 11 Pattern Recognition Lab, Friedrich-Alexander University, Erlangen-Nürnberg, Erlangen, Germany

**Keywords:** Cognitive ageing, Diagnostic markers

## Abstract

Action-concept outcomes are useful targets to identify Parkinson’s disease (PD) patients and differentiate between those with and without mild cognitive impairment (PD-MCI, PD-nMCI). Yet, most approaches employ burdensome examiner-dependent tasks, limiting their utility. We introduce a framework capturing action-concept markers automatically in natural speech. Patients from both subgroups and controls retold an action-laden and a non-action-laden text (AT, nAT). In each retelling, we weighed action and non-action concepts through our automated Proximity-to-Reference-Semantic-Field (P-RSF) metric, for analysis via ANCOVAs (controlling for cognitive dysfunction) and support vector machines. Patients were differentiated from controls based on AT (but not nAT) P-RSF scores. The same occurred in PD-nMCI patients. Conversely, PD-MCI patients exhibited reduced P-RSF scores for both texts. Direct discrimination between patient subgroups was not systematic, but it yielded best outcomes via AT scores. Our approach outperformed classifiers based on corpus-derived embeddings. This framework opens scalable avenues to support PD diagnosis and phenotyping.

## Introduction

The quest for cognitive markers of Parkinson’s disease (PD) highlights the usefulness of assessing action concepts—mainly verbs denoting bodily movement, such as *run*, *jump*, *applaud*, and *dance*^1^. Indeed, their processing hinges on motor brain networks^2–4^ and is influenced by the speed and precision of bodily actions^5,6^. Since PD compromises these neural circuits and behavioral dimensions, action concepts have been proposed as a robust target to identify patients and differentiate between phenotypes^1^. However, most evidence comes from burdensome, examiner-dependent, non-ecological tasks, limiting the framework’s sensitivity, scalability, and clinical utility^1,7–9^. To overcome such caveats, this machine learning study leverages automated semantic analysis of action and non-action stories by healthy controls (HCs) and early PD patients, including subgroups with and without mild cognitive impairment (PD-MCI, PD-nMCI).

Affecting over 6 million people, PD is the most prevalent and fastest-growing movement disorder worldwide^10^. Patients are typified by primary motor impairments and diverse cognitive symptoms, mainly linked to frontostriatal degeneration^11^. Despite their usefulness, gold-standard clinical, imaging, and biospecimen tests often prove invasive, costly, and/or unspecific to reveal disease-specific signatures^11^. A thriving complement comes from tasks tapping action concepts, as defined above. Indeed, in varied PD cohorts, this approach has revealed early, category-specific, and disease-differential deficits^9,12^, related to canonical brain alterations^9,13,14^ and sensitive to medication status^15^—for a review, see Birba et al.^1^. Moreover, assessments of action concepts can robustly discriminate between persons with PD-MCI and PD-nMCI: whereas action-concept deficits occur alongside non-action concept deficits in the former group, they prove selective in PD-nMCI patients^7,16^, who account for roughly three-fourths of the population^17–19^.

Yet, most evidence comes from highly controlled tasks that prove lengthy (lasting up to 25 min), unnatural (e.g., requiring fast decisions over random sequences of context-free stimuli), and/or based on fallible examiner-dependent scoring^1^. Promisingly, incipient machine learning studies indicate that automated capturing of action semantics in (semi)spontaneous discourse can identify early-stage PD patients and discriminate between subgroups (e.g., based on dopamine bioavailability) in a naturalistic, objective, cost-effective, and patient-friendly setting^8,20^. However, none of these reports employed a paradigm *strategically designed* to elicit action concepts in unfolding speech, let alone while exploring their sensitivity to distinct disease phenotypes. A fruitful path for clinical PD research thus emerges at the crossing of behavioral neurology, cognitive neuroscience, and natural language processing.

**Fig. 1:**
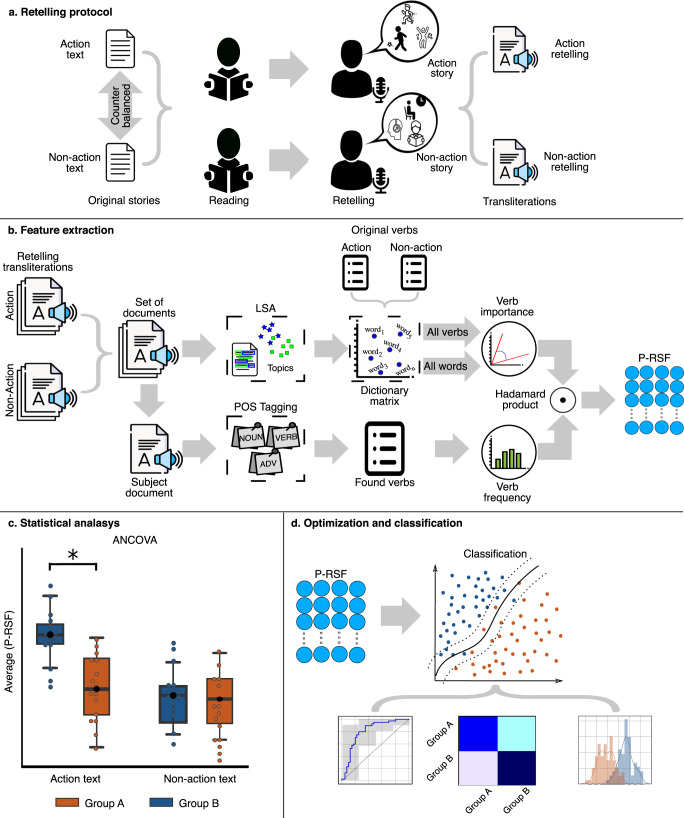
Analysis pipeline. **a** Participants read and immediately retold an AT and an nAT. **b** P-RSF scores were extracted from each subject’s AT and nAT retelling. **c** Statistical between-group comparisons were made via ANCOVAs, covarying for MoCA and IFS scores. **d** Classification analyses were based on support vector machines, with results represented via receiver operating characteristic (ROC) curves, confusion matrices, and distribution plots of P-RSF scores. These analyses were applied to discriminate between (i) all PD patients and all HCs, (ii) PD-nMCI patients and HCs, (iii) PD-MCI patients and HCs, and (iv) PD-nMCI and PD-MCI patients. AT action text, nAT non-action text, ANCOVA analysis of covariance, MoCA Montreal Cognitive Assessment, IFS INECO Frontal Screening, P-RSF Proximity-to-Reference-Semantic-Field, PD Parkinson’s disease, PD-MCI Parkinson’s disease with mild cognitive impairment, PD-nMCI Parkinson’s disease without mild cognitive impairment.

Here we examined whether automated discourse-level analysis of action semantics can (i) identify PD patients in a cognitively heterogeneous cohort and (ii) differentiate between PD-MCI and PD-nMCI individuals. Early-stage patients and HCs read and immediately retold two matched, validated stories: an action text (AT, rich in movement descriptions) and a non-action text (nAT, focused on non-motoric events)^4,7–9,21,22^. For each text, we extracted semantic features via latent semantic analysis (LSA) and implemented a Proximity-to-Reference-Semantic-Field (P-RSF) metric, capturing the weight of action and non-action concepts across retold texts. We then used inferential statistical models (ANCOVAs, controlling for cognitive symptom severity) and support vector machine (SVM) classifiers to assess whether patients and HCs could be discriminated via semantic information. Moreover, we performed additional analyses based on corpus-derived word embeddings as a benchmark to gauge the robustness of our metric. Finally, exploratory correlations were performed between P-RSF scores and an index of motor symptom severity. The pipeline is depicted in Fig. 1.

We advanced three hypotheses and an exploratory question. First, in line with the literature^1^, we predicted that patients in the overall cohort would be robustly identified through AT (but not nAT) retelling (i.e., via action semantic fields). Second, building on the previous work^7,16^, we hypothesized that such a selective AT pattern would be replicated in the PD-nMCI subgroup. Third, considering the same antecedents^7,16^, we anticipated that PD-MCI patients would be discriminated through semantic patterns in either text. Finally, we inquired whether any text could directly discriminate between PD-nMCI and PD-MCI patients. By testing these predictions, we aim to open new objective, affordable, and ecological avenues towards scalable markers of PD.

## Results

### All PD patients vs. all HCs

**Fig. 2:**
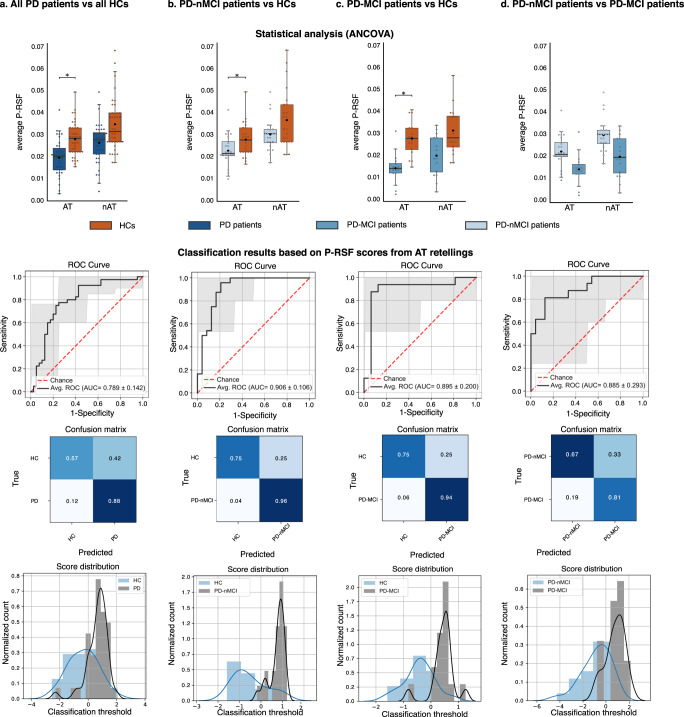
Statistical comparison between groups in each tandem (top insets) and classification results using action texts (lower insets). **a** All PD patients vis-à-vis all HCs. **b** PD-nMCI patients vis-à-vis HCs. **c** PD-MCI patients vis-à-vis HCs. **d** PD-nMCI vis-à-vis PD-MCI patients. AT action text, nAT non-action text, P-RSF Proximity-to-Reference-Semantic-Field, PD Parkinson’s disease, PD-MCI Parkinson’s disease with mild cognitive impairment, PD-nMCI Parkinson’s disease without mild cognitive impairment.

Comparisons between all PD patients and all HCs (Fig. 2a, top inset) revealed significantly lower P-RSF values for the former group in the AT [*F*(1,76) = 10.55, *p* = 0.002, η_p_^2^ = 0.12], alongside non-significant between-group differences in the nAT [*F*(1,76) = 2.92, *p* = 0.092, η_p_^2^ = 0.03]. Similarly, classification between PD patients and HCs was robust for the AT (AUC = 0.80, accuracy = 72.5%) and near chance for the nAT (AUC = 0.60, accuracy = 58.8%)—Table 1 and Fig. 2a (lower insets).

**Table 1:** Machine learning results for each group tandem based on the P-RSF metric.

	Accuracy	Sensitivity	Specificity	*F*-score
*All PD patients vs. all HCs*				
Action text	72.5	80.0	65.0	72.2
Non-action text	58.8	57.5	60.0	57.7
*PD-nMCI patients vs. HCs*				
Action text	85.0	96.7	73.3	84.2
Non-action text	48.3	41.6	55.0	41.8
*PD-MCI patients vs. HCs*				
Action text	82.5	95.0	70.0	78.7
Non-action text	72.5	75.0	70.0	66.7
*PD-nMCI vs. PD-MCI patients*				
Action text	69.5	75.0	65.0	67.6
Non-action text	61.5	60.0	63.3	58.7

*HCs* healthy controls, *PD* Parkinson’s disease, *PD-nMCI* Parkinson’s disease without mild cognitive impairment, *PD-MCI* Parkinson’s disease with mild cognitive impairment.

### PD-nMCI patients vs. HCs

Compared with HCs, PD-nMCI patients exhibited lower P-RSF scores in the AT [*F*(1,44) = 4.27, *p* = 0.04, η_p_^2^ = 0.08], but not in the nAT [*F*(1,44) = 3.387, *p* = 0.072, η_p_^2^ = 0.07]—Fig. 2b, top panel. Classification between participants in these groups was successful upon considering P-RSF values from the AT (AUC = 0.93, accuracy = 85%), whereas the nAT yielded chance-level outcomes (AUC = 0.55, accuracy = 48.3%)—Table 1 and Fig. 2b (lower insets).

### PD-MCI patients vs. HCs

P-RSF values in the AT were also higher for PD-MCI patients than for HCs [*F*(1,28) = 4.47, *p* = 0.04, η_p_^2^ = 0.14], there being no significant group differences in the nAT [*F*(1,28) = 0.69, *p* = 0.414, η_p_^2^ = 0.02] –Fig. 2c, top inset. In this tandem, good classification scores were obtained for both the AT (AUC = 0.90, accuracy = 82.5%) and the nAT (AUC = 0.80, accuracy = 72.5%)—Table 1 and Fig. 2c (lower insets).

### PD-nMCI vs. PD-MCI patients

Comparisons of P-RSF scores between both patient groups revealed non-significant differences for the AT [*F*(1,36) = 1.69, *p* = 0.20, η_p_^2^ = 0.05] and the nAT [*F*(1,36) = 0.007, *p* = 0.93, η_p_^2^ = 0.01]—Fig. 2d, top inset. Yet, classification results were more robust for the AT (AUC = 0.82, accuracy = 69.5%) than for the nAT (AUC = 0.53, accuracy = 61.5%)—Table 1 and Fig. 2d (lower insets).

### Classification based on corpus-derived verb-to-verb semantic distance

Classification outcomes based on GloVe-derived distances between participants’ verbs and those in the original stories were systematically lower than those obtained with the P-RSF metric (Table 2). This was true for the classification of all PD patients vs. all HCs (AT: AUC = 0.61, accuracy = 46.8%; nAT: AUC = 0.57, accuracy = 57.4%), PD-nMCI patients vs. HCs (AT: AUC = 0.62, accuracy = 59.2; nAT: AUC = 48.6, accuracy = 52.5%), PD-MCI patients vs. HCs (AT: AUC = 0.58, accuracy = 57.5; nAT: AUC = 0.75, accuracy = 67.5), and PD-nMCI vs. PD-MCI patients (AT: AUC = 0.59, accuracy = 63.3%; nAT: AUC = 0.59, accuracy = 60.5%).

**Table 2:** GloVe embeddings: classification based on corpus-derived verb-to-verb semantic distance.

	Accuracy	Sensitivity	Specificity	*F*-score
*All PD patients vs. all HCs*				
Action text	46.8	88.3	29.0	44.6
Non-action text	57.4	45.0	62.7	51.4
*PD-nMCI patients vs. HCs*				
Action text	59.2	65.0	53.3	55.7
Non-action text	52.5	28.3	76.7	47.5
*PD-MCI patients vs. HCs*				
Action text	57.5	30.0	85.0	48.7
Non-action text	67.5	55.0	80.0	61.7
*PD-nMCI vs. PD-MCI patients*				
Action text	63.3	20.0	90.0	48.0
Non-action text	60.5	40.0	71.7	50.3

*HCs* healthy controls, *PD* Parkinson’s disease, *PD-nMCI* Parkinson’s disease without mild cognitive impairment, *PD-MCI* Parkinson’s disease with mild cognitive impairment.

### Classification based on overall semantic structure

Classification outcomes based on GloVe word embeddings were also lower than those obtained with the P-RSF metric (Table 3). This was true for the classification of all PD patients vs. all HCs (AT: AUC = 0.54, accuracy = 60.62%; nAT: AUC = 0.57, accuracy = 62.06%), PD-nMCI patients vs. HCs (AT: AUC = 0.68, accuracy = 65%; nAT: AUC = 0.63, accuracy = 62.5%), PD-MCI patients vs. HCs (AT: AUC = 0.65, accuracy = 67.5%; nAT: AUC = 0.78, accuracy = 67.5%), and PD-nMCI vs. PD-MCI patients (AT: AUC = 0.63, accuracy = 59.3%; nAT: AUC = 0.68, accuracy = 66.5%).

**Table 3:** GloVe embeddings: classification based on overall semantic structure.

	Accuracy	Sensitivity	Specificity	*F*-score
*All PD patients vs. all HCs*				
Action text	60.62	53.33	64.67	54.78
Non-action text	62.06	46.67	68.00	54.19
*PD-nMCI patients vs. HCs*				
Action text	65.00	65.00	65.00	62.45
Non-action text	62.50	68.33	56.67	56.87
*PD-MCI patients vs. HCs*				
Action text	67.50	60.00	75.00	60.67
Non-action text	67.50	60.00	75.00	64.33
*PD-nMCI vs. PD-MCI patients*				
Action text	59.33	25.00	83.33	44.15
Non-action text	66.50	50.00	80.00	60.14

*HCs* healthy controls, *PD* Parkinson’s disease, *PD-nMCI* Parkinson’s disease without mild cognitive impairment, *PD-MCI* Parkinson’s disease with mild cognitive impairment.

### Exploratory correlation analyses

Exploratory correlation analyses revealed that P-RSF scores from the AT and the nAT were not significantly associated with UPDRS-III scores in any group (Table 4).

**Table 4:** Exploratory correlations between P-RSF scores and UPDRS-III scores.

	Correlations between P-RSF scores on the AT and UPDRS-III scores		Correlations between P-RSF scores on the nAT and UPDRS-III scores	
	*r*-value	*p*-value	*r*-value	*p*-value
All PD patients	−0.171	0.29^a^	−0.163	0.31^b^
PD-nMCI patients	0.147	0.49^a^	0.029	0.89^b^
PD-MCI patients	−0.056	0.84^b^	−0.184	0.49^a^

*DUPDRSIII* part III of the Unified Parkinson’s disease Rating Scale, *PD* Parkinson’s disease, *PD-nMCI* Parkinson’s disease without mild cognitive impairment, *PD-MCI* Parkinson’s disease with mild cognitive impairment.

^a^Based on Pearson’s correlations, given the data distribution.

^b^based on Spearman’s correlations, given the data distribution.

## Discussion

We developed an automated framework to capture semantic markers of PD and its cognitive phenotypes through AT and nAT retelling. The weight of action and non-action concepts in each retold story was quantified with our P-RSF metric, compared between groups through ANCOVAs, and used to classify between patients and HCs via machine learning. P-RSF scores from AT (but not nAT) retelling robustly discriminated between PD patients and HCs. Subgroup analyses replicated this pattern in PD-nMCI patients but not in PD-MCI patients, who exhibited reduced P-RSF scores for both AT and nAT retellings. Also, though not systematic, discrimination between PD-nMCI and PD-MCI was better when derived from AT than nAT retellings. Moreover, our approach outperformed classifiers based on corpus-derived word embeddings. Finally, no significant associations emerged between P-RSF and UPDRS-III scores. These findings have translational implications, as discussed next.

Comparisons between the overall PD and HC groups revealed significantly lower P-RSF scores for the AT in the patients, with non-significant differences for the nAT. This points to a selective impariment in evoking action-related events, as previously observed through lexical decision^12^, semantic similarity judgment^12^, picture naming^13^, and text comprehension^9^ tasks. Of note, present results were covaried for MoCA and IFS outcomes as indices of cognitive symptom severity. This replicates the finding that action-concept deficits in PD^7^ and other disorders with motor-network disruptions^4^ are not driven by domain-general cognitive dysfunctions, but rather constitute *sui generis* disturbances.

In the same vein, classification between patients and HCs via P-RSF scores was robust for AT retellings (AUC = 0.80, accuracy = 72.5%) and near-chance for nAT retellings. While previous machine learning studies on PD have reported action-concept alterations in (semi)spontaneous discourse^8,20^, our study shows their *selective* occurrence relative to non-action semantic fields. Such a pattern supports the disrupted motor grounding hypothesis, which posits that if action concepts distinctly recruit motor mechanisms in HCs^2,3^, then they should be differentially impaired in persons with motor-system disruptions^1^. Indeed, action-concept processing in PD has been linked to alterations in regions subserving movement initiation and observation, such as the primary motor cortex^14^ and the extrastriate body area^9^, which are distinctively compromised in this population^23^. Our approach offers new possibilities towards the probabilistic detection of persons with PD.

Yet, action-semantic measures may not be equally sensitive across the disease’s cognitive phenotypes. Reduced P-RSF scores for AT retelling were selective only in PD-nMCI patients. In this subgroup, subject-level classification increased substantially (AUC = 0.93, accuracy = 85%), contrasting with the chance-level classification obtained through nAT outcomes. Contrariwise, PD-MCI patients were robustly discriminated from HCs based on P-RSF scores from *both* AT and nAT retelling. This aligns with text comprehension^7^ and picture-naming^16^ studies showing that action-concept deficits emerge selectively in PD-nMCI but are accompanied by non-action-concept impairments in PD-MCI. Such evidence reinforces the distinct link between action-concept processing and motor-system impairment in PD: in mainly motoric phenotypes (i.e., PD-nMCI), we propose, AT retelling becomes distinctly compromised, arguably due to the distinct reliance of action concepts on more focally compromised motor mechanisms^9,13,14^. Conversely, when patients’ motoric deficits are accompanied by widespread cognitive disturbance (i.e., PD-MCI), diverse semantic fields would become affected, arguably because multimodal conceptual processing recruits diverse brain regions that support myriad cognitive functions^2,24^ and which may be specifically atrophied in PD-MCI^25^. Although the neural signatures of semantic processing differences between PD-nMCI and PD-MCI remain poorly understood, this conjecture aligns with present and previous findings, paving the way for new investigations.

Yet, direct contrasts between patient subgroups yielded less consistent results. On the one hand, ANCOVAs failed to reveal significant differences in either text. However, P-RSF scores from the AT surpassed those from the nAT in classifying patients with and without MCI, with above-chance accuracy (69.5%) and a solid AUC value (0.82). Thus, our approach may prove more sensitive to discriminate between phenotypes in probabilistic subject-level terms than at the group level. Previous discourse-level evidence indicates that action-concept measures can discriminate between PD patients on and off medication^8^. Though inconclusive, our study suggests that examinations of this domain may also be worth pursuing to discriminate between patients with different cognitive profiles. This would be a relevant effort, since standard screening instruments, such as the Mini-Mental State Examination, are bound to ceiling effects and often fail to capture cognitive dysfunction in PD^26^. Given that dementia symptoms may be unnoticed in over half of PD patients^27^, our semantic framework may be combined with other approaches, such as motor speech assessments^28^, to establish phenotypic distinctions within the overall patient population.

Also, the P-RSF metric systematically outperformed classifiers based on GloVe embeddings. First, this was the case when such embeddings were used to calculate distance between verbs in the retellings and in the original texts. This is likely so because P-RSF uses LSA, yielding vectors based on our original texts’ bag of words. As such, our approach considers the frequency and co-occurrence patterns of the AT and the nAT, systematically designed to capture the action vs. non-action opposition while being controlled for over 20 psycholinguistic variables^9^. Conversely, GloVe embeddings result from a model trained with a large corpus that targets no specific hypothesis-driven semantic category. Thus, words’ semantic spaces are created by reference to multiple topics rather than predefined semantic fields informed by previous findings.

Moreover, the P-RSF metric offered better classification than analyses based on the texts’ overall semantic structure (also obtained via GloVe). This reinforces the view that semantic abnormalities in PD are mainly driven by action concepts. Indeed, while PD patients are consistently affected in this category^1^, they evince no major alterations in more general semantic measures, including processing of abstract^12^ and social concepts^9^, semantic granularity^29^, and ongoing semantic variability^29^, among others. Note, also, that the P-RSF metric allows identifying specific semantic memory domains that are compromised and spared, favoring interpretability. Taken together, these observations attest to the distinct usefulness of our methodological framework.

Finally, exploratory correlation analyses for each text in each group revealed non-significant associations between P-RSF scores and UPDRS-III scores. This suggests that patients’ action semantic alterations were not proportional to their degree of motor impairment. This finding replicates previous studies reporting null associations between UPDRS-III scores and performance in other action-concept tasks, including lexical decision^30^, picture naming^16^, verb generation^31^, and action fluency^32^. Tentatively, this suggests that semantic abnormalities in PD hold irrespective of motor symptom severity, reinforcing the critical role of *cognitive dysfunction* in determining whether concept-level alterations are confined to the action domain or general to other semantic categories^7,16^.

It is worth stressing that present results were obtained with naturalistic tasks and automated methods. Action-semantic deficits are well-established in the PD literature^1^, but they are typically captured through burdensome tasks that are rarely, if ever, found in real life. For example, participants have been asked to decide whether successive letter strings constitute real words^12^, name or associate decontextualized pictures^16^, or press buttons with particular hand positions after sentence listening^33^. Such settings may prove tiring, frustrating, and cognitively taxing, compromising data quality, task completion, and ecological validity. Moreover, performance in several relevant tasks, such as fluency^34^ and picture naming^16^, is established by examiners, who must single-handedly decide whether each response meets correctness criteria. Ensuing scores may thus be prone to inter-rater variability, potentially undermining reliability. Automated analysis of free speech overcomes these issues, offering a patient-friendly, ecologically valid, and objective framework to collect clinically usable data. In particular, our approach, rooted in a strategic task and a theory-driven metric, combines the sensitivity of action-semantic assessments for PD with the clinical potential of automated discourse analysis. Further work in this direction could hone the translational relevance of linguistic assessments in the quest for early markers of PD.

Our study is not without limitations. First, our sample size was moderate, especially in the subgroup analyses. Although previous natural discourse studies on PD^8,20,35^ and other neurodegenerative disorders^29^ have yielded robust results with similar and smaller groups, replications with more participants would be needed. Relatedly, results stemmed from the distance between the original texts’ verbs and the ones produced by participants in each training fold, meaning that they might change if new participants were tested and produced verbs that were not present in such folds. Hence, our models should be enriched with larger samples (ideally allowing for out-of-sample validation) so as to strengthen their generalizability. Second, the AT and nAT we employed described only a few action and non-action events which may not be directly relevant to patients’ daily activities. This should be circumvented in future studies, aiming for greater ecological validity. Third, the retelling task taxes working memory resources. Although statistical results were controlled for measures of cognitive (including memory) function, this might partly influence overall task conditions for PD-MCI patients. Future studies could harness our approach with tasks that reduce working memory demands, such as online descriptions of action and non-action pictures. Fourth, our study was restricted to Spanish, precluding insights on cross-linguistic generalizability. As argued recently^36^ and as done in other PD studies^35^, replications over typologically different languages would be important to ascertain the external validity of these results. Finally, as in recent text comprehension research^9^, further studies could include neural measures to reveal anatomo-functional signatures of the different behavioral profiles reported in each group.

In conclusion, well-established semantic markers of PD can be captured automatically in connected discourse. In particular, disruptions in the construal of action concepts seem useful to identify persons with PD and to detect patterns that differ between those with impaired and spared cognitive skills. Given its objectivity, low cost, and scalability, this approach can fruitfully complement mainstream approaches to characterizing, phenotyping, and diagnosing patients. Computerized language analysis, thus, represents a promising tool towards richer clinical research on this population.

## Methods

### Participants

The study involved 80 Spanish speakers from a well-characterized cohort^7,28^, including 40 early PD patients with varied cognitive profiles and 40 HCs. This sample size matches or surpasses that of previous PD studies using automated language tools^8,20^. All participants were Hispanics/Latinos from Colombia, self-identified as white in terms of race. No participant reported a multi-racial background nor indigenous, Asian, or African ancestry. Patients were diagnosed based on United Kingdom PD Society Brain Bank criteria^37^, with motor assessments via the UPDRS-III^38^ and the Hoehn & Yahr scale^39^, and executive function testing through the INECO Frontal Screening (IFS) battery^40^. No patient had primary language deficits, signs of Parkinson-plus, deep brain stimulation antecedents, or concomitant neurological, psychiatric, or addiction disorders. Results from the Barthel Index^41^ and the Lawton & Brody Index^42^ indicated that all patients were functionally independent.

MCI screening followed level-1 criteria of the Movement Disorder Society Task Force^43^, including the Montreal Cognitive Assessment (MoCA)^44^, a sensitive tool for PD^26^. Patient sub-groups were formed based on region-specific MoCA cutoffs^45^, as in the previous works^7,28^. Those with normal MoCA scores integrated the PD-nMCI group (*n* = 24), while those with MoCA scores below the MCI cutoff were classified as PD-MCI (*n* = 16)—Supplementary Information 1. These sample sizes are similar to or larger than those of previous machine learning studies^8,28,46^.

HCs had functional independence, normal MoCA scores, and no history of neurological or psychiatric disease. They were sociodemographically matched with patients in the overall cohort (Table 5) as well as in each sub-group (Supplementary Information 1). Patient sub-groups were also matched for sociodemographic and clinical variables. All patients were tested during the “on” phase of anti-parkinsonian medication, converted to Levodopa equivalent daily dose^47^.

**Table 5:** Participants’ demographic and clinical data.

	All PD patients (*n* = 40)	All HCs (*n* = 40)	All PD patients vs. all HCs
*Sociodemographic variables*			
Sex (F:M)	15:25	15:25	–
Age	62.25 (9.27)	61.87 (7.32)	0.84^a^
Years of education	12.23 (5.01)	12.75 (4.62)	0.63^a^
*Clinical variables*			
Years since diagnosis	5.65 (3.72)	–	–
UPDRS-III	31.0 (12.54)	–	–
H&Y	2.05 (0.29)	–	–
IFS battery	19.56 (3.36)	22.92 (2.67)	0.07^a^
Barthel Index	100 (0)	–	–
L&B	8 (0)	–	–
MoCA	24.75 (2.99)	26.7 (1.63)	**>0.01**^a^
LED	658.27 (375)	–	–

Data presented as mean (*SD*); sex was self-reported.

*PD* Parkinson’s disease, *HCs* healthy controls, *UPDRS-III* Unified Parkinson’s Disease Rating Scale, part III, *H&Y* Hoehn & Yahr scale, *IFS* INECO Frontal Screening, *L&B* Lawton & Brody Index, *MoCA* Montreal Cognitive Assessment, *LED* Levodopa equivalent dose.

^a^*p*-values calculated using Mann–Whitney *U* tests.

Participants provided written informed consent pursuant to the Declaration of Helsinki. The study was approved by the Institutional Ethics Committee of Antioquia University (resolutions 14-10-569 and 15-10-569).

### Materials

The AT and the nAT were created through a systematic protocol used in previous action semantics research^4,7,9,21,48^. The former story focused on the characters’ bodily movements, including single-limb and whole-body actions performed in isolation or during interactions with objects and other people (e.g., *Johnny ran quickly to the place where the clown was jumping and dancing*). This text offered several locative and temporal specifications, alongside details of how bodily actions were performed. Conversely, the nAT foregrounded its characters’ feelings, thoughts, and perceptions (e.g., *Albert was euphoric*), without explicit mention of bodily actions. Abundant circumstantial information was included about the places, objects, emotions, and internal states involved in the story.

Each text was based on the same 22 grammatical patterns, pseudo-randomly distributed in each case. Selected lexical items were chosen to compose each story. These included 32 verbs per text, chosen based on semantic, syntactic, and distributional criteria to operationalize the action/non-action distinction. The stories were matched for character count; overall and content-word-type counts; mean content-word frequency, familiarity, syllabic length, graphemic length, and imageability; sentence and sentence-type counts; reading difficulty; grammatical correctness, coherence, and comprehensibility; readability rating; and emotional content (Table 6). Both texts communicated mostly literal meanings and contained no jargon (for full transcriptions and English translations, see Supplementary Information 2).

**Table 6:** Linguistic features of the stories.

	Action text	Non-action text	Statistic	*p-*value*
Characters^a^	944	978	*χ²* = 0.60	0.44
Words	208	204	*χ²* = 0.04	0.84
Nouns	48	44	*χ²* = 0.17	0.68
Adjectives	7	9	*χ²* = 0.25	0.62
Adverbs	6	8	*χ²* = 0.29	0.59
Verbs	**32**	**32**	*χ²* = 0	**1**
Action verbs	**1**	**24**	*χ²* = 21.16	**<0.001**
Non-action verbs	**31**	**8**	*χ²* = 13.56	**<0.001**
Content word frequency^b^	1.63	1.79	*t* = 1.53	0.13
Content word familiarity^b^	6.15	6.24	*t* = 0.74	0.46
Content word imageability^c^	5.25	4.97	*t* = 1.39	0.17
Content word syllabic length^c^	2.52	2.49	*t* = 0.25	0.80
Content word graphemic length^c^	6.16	6.26	*t* = 0.36	0.72
Sentences	22	22	*χ²* = 0	1
Minor sentences	3	3	*χ²* = 0	1
Simple sentences	8	8	*χ²* = 0	1
Compound sentences	4	3	*χ²* = 0.14	0.71
Complex/complex-compound sentences	7	8	*χ²* = 0.07	0.80
Grammatical correctness^d^	4.52	4.29	*t* = 0.66	0.51
Coherence^d^	4.05	3.86	*t* = 0.62	0.54
Comprehensibility^d^	4.24	4.10	*t* = 1.05	0.30
Readability^e^	79.92	77.3	*χ²* = 0.04	0.83
Reading difficulty^f^	Fairly easy	Fairly easy	–	–
Emotional valence^g^	33.25	33.29	*F* = 1.0	0.33
Arousal^g^	4.81	4.24	*F* = 2.7	0.20

^a^Character count was performed without counting spaces.

^b^Data extracted from the LEXESP database, through B-Pal^47^ (results based on the mean of all content words in each text).

^c^Data extracted from B-Pal^47^ (results based on the mean of all content words in each text);

^d^Data collected from a panel of 10 Spanish-speaking undergraduates, based on Likert scales ranging from 1 (very low) to 5 (very high).

^e^Based on the Szigriszt-Pazos Index^48^.

^f^Based on the Inflesz scale rating^49^.

^g^Data collected from a panel of 17 native Spanish speakers, who rated each sentence in the texts in terms of overall emotional content (positive, negative, or neutral) and arousal level for positive and negative emotions (based on seven-point Likert scales).

### Procedure

Participants sat at a desk in a quiet room, wearing a noise-canceling headband microphone (Genius HS-04SU) connected to a laptop. First, for familiarization purposes, they were handed a practice text and asked to read it silently, at their own pace. Once finished, the page was removed and they were asked to retell the story in their own words, with normal pace, cadence, and volume. The same procedure was then followed for the AT and the nAT, whose order was counterbalanced across participants (Fig. 1a). All participants retold both texts. Audio files were deidentified, recodified, and saved via WaveSurfer 1.8.8p4 (.wav, 44100 Hz, 16 bits). Recordings were transcribed via an automatic speech-to-text service and manually revised following standard criteria from the Royal Spanish Academy, as in previous works^20,35^. Transcripts were fully faithful to the patients’ production: grammatical mistakes, false starts, hesitations, and other speech infelicities were left unedited for analysis^20,35^.

### Data preprocessing and feature extraction

Following standard preprocessing approaches, all words in each transcript were converted to lowercase letters. Accents, numbers, punctuation signs, and stop words were then removed, and the remaining words were lemmatized^29^. Feature extraction was performed for AT retellings and nAT retellings separately. This yielded two corpora, each with its own vocabulary (i.e., a list of unique words for the ATs and another one for the nATs). Three main steps were applied thereon: (i) computation of words’ vector representations; (ii) estimation of the verbs’ importance in each original story; and (iii) calculation of the P-RSF metric, capturing the weight of action and non-action semantic fields across in each retelling (Fig. 1b).

In each type of retelling separately, vector representations were obtained for each word via LSA –a method that represents each document based on latent features or topics, previously used in PD research^20^. First we constructed a document-term matrix. The cardinality of this matrix is *m* × *v*, where *m* is the number of documents in the corpus and *v* is the number of words in the vocabulary. The matrix was estimated using the Bag-of-Words model, which computes the vector representation of a document based on the frequency of each of its constituting words^49^. As in previous automated semantic analysis on PD^20^, the document-term matrix was further processed via singular value decomposition (SVD) to obtain two matrices, namely: an encoding and a dictionary matrix. The encoding matrix relates each document with its weight in each topic, and the dictionary matrix relates each term or word in the vocabulary with its weight in each topic. The topics considered for analyses were those that accumulated 95% of the explained variance^50^. The dictionary matrix was then used to create a vector representation for each word in the vocabulary of the AT, on the one hand, and of the nAT, on the other.

We then computed the importance of the verbs in each retelling, exclusively targeting those that also appeared in the original stories. This yielded 27 verbs for the AT (24 of which denoted physical actions) and 23 for the nAT (20 of which evoked no physical actions) –mean motility ratings^51^ were 3.91 (SD = 1.30) for AT verbs and 2.29 (*SD* = 0.79) for nAT verbs. Then, for each story, we calculated the similarity among each original verb and all words in each corpus via the average cosine distance. We thus obtained a verb importance measure, namely, the weight of the semantic field of each original verb in the semantic field generated by the words in the retellings.

Finally, we used a part-of-speech-tagger to find all verbs in each text set^52^, and computed the occurrence frequency of each original verb in each retelling. When a verb from a retelling did not correspond to any original verb, its occurrence frequency was estimated as the distance to the closest original verb via cosine similarity. Then, an occurrence matrix was derived from these vector representations in each retelling document. The cardinality of this matrix was *m* × *v*, where *m* is the number of documents and *n* is the number of original verbs. The P-RSF matrix was then estimated using the Hadamard product (i.e., element-wise product) between the occurrence matrix and the verb importance vector—the lower the P-RSF value, the lower the P-RSF value, the lower the weight of the target (action or non-action) concepts in a retelling. This matrix was used for inferential analyses (via ANCOVAs) and as a feature matrix for machine learning analyses.

### Statistical analysis

The features described above were statistically compared between groups in each tandem, namely: (a) all PD patients and all HCs, (b) PD-nMCI patients and HCs, (c) PD-MCI patients and HCs, and (d) PD-nMCI and PD-MCI patients (Fig. 1c). In each case, mean P-RSF scores on each text were compared between groups via one-way ANCOVAs, covarying for MoCA and IFS scores as measures of cognitive symptom severity –as in previous action semantic studies on PD^7^ and other neurological disorders^4^. Alpha levels were set at *p* < 0.05. Effect sizes were calculated through partial eta squared (η_p_^2^) tests. All statistical analyses were performed on Pingouin, an open-source statistical package^53^.

### Machine learning analyses

The semantic features were also used to classify between participants in each group tandem: (a) all PD patients and all HCs, (b) PD-nMCI patients and HCs, (c) PD-MCI patients and HCs, and (d) PD-nMCI and PD-MCI patients. In each case, individual binary classifiers were run for the AT and for the nAT. These analyses employed SVM with a Gaussian kernel, a classifier that has proven robust in experiments with similar data^28,54^ and moderate sample sizes^55,56^. The kernel bandwidth and regularization parameters were optimized through a randomized search strategy that avoids overfitting and guarantees generalization^57^. Instead of searching over the entire grid of possible values of hyper-parameters, the randomized search only evaluates a random sample of points on the grid. The models were trained following a participant-independent nested five-fold cross-validation strategy, with four folds used internally for hyper-parameter optimization based on training set outcomes^28,54^. That is, in each iteration, four folds were used to train/optimize the model’s meta-parameters and the remaining fold was used for testing (thus, in each iteration, each participant’s vectors were used for either training or testing, but not for both). Main results for each tandem were represented via an area under the ROC curve plot, a confusion matrix, and a distribution plot (Fig. 1d).

In addition, we implemented two complementary approaches as benchmarks to ascertain the discriminatory utility of our approach. First, we explored classifier performance when verbs’ semantic distance was established by reference to corpus-derived embeddings, as in previous PD research^8^. To this end, we used Global Vectors for Word Representation (GloVe), a method that captures linear substructures of a text’s word vector space based on summated statistics of the co-occurrence between any two words in a corpus^58^. The same part-of-speech-tagger used in our main analyses was employed to find all verbs in each preprocessed retelling. Then, the numerical representation of all verbs in each retelling was obtained using a previously reported GloVe model, pre-trained with the Wikipedia 2018 Corpus, which contains ≈709 million Spanish words^59^. We computed the cosine distance between each verb in the retelling and the verbs in the original story (i.e., the same verbs used in our main analyses). The feature vector of each retelling was computed as the mean distance of all verbs in the retelling to each verb in the corresponding original story. Second, we examined classifier performance based on each retelling’s overall semantic structure, as captured by GloVe embeddings. Numerical representations were obtained for all post-tagged words in each processed retelling. The overall feature vector of each retelling was calculated as the mean word embedding of all its words. In both approaches, classification models were created using support vector machines, with the same cross-validation strategy used in our main analyses.

### Exploratory correlation analyses

Finally, to examine the relation between action concept processing and motor symptom severity, we performed exploratory analyses between P-RSF scores and UPDRS-III scores These analyses were in each patient group, for each text separately, using Pearson’s or Spearman’s correlations depending on data distribution.

### Reporting summary

Further information on research design is available in the Nature Research Reporting Summary linked to this article.

## Supplementary information

Supplementary information

Reporting Summary

## Data Availability

The datasets generated and/or analyzed during the current study are available in the Open Science Framework (OSF) repository under the title “García (2022). Semantics of retelling in PD”, https://osf.io/6xc5b/, 10.17605/OSF.IO/6XC5B.

## Code availability

Code used in this study will be made available upon reasonable request.

## References

[1] Birba A (2017). Losing ground: frontostriatal atrophy disrupts language embodiment in Parkinson’s and Huntington’s disease. Neurosci. Biobehav. Rev..

[2] Pulvermüller F (2013). How neurons make meaning: brain mechanisms for embodied and abstract-symbolic semantics. Trends Cogn. Sci..

[3] García AM (2019). How meaning unfolds in neural time: embodied reactivations can precede multimodal semantic effects during language processing. NeuroImage.

[4] Moguilner S (2021). Multimodal neurocognitive markers of frontal lobe epilepsy: insights from ecological text processing. NeuroImage.

[5] García AM, Ibáñez A (2016). A touch with words: dynamic synergies between manual actions and language. Neurosci. Biobehav. Rev..

[6] Cervetto S (2021). The neural blending of words and movement: event-related potential signatures of semantic and action processes during motor–language coupling. J. Cogn. Neurosci..

[7] García AM (2018). Parkinson’s disease compromises the appraisal of action meanings evoked by naturalistic texts. Cortex.

[8] Norel R (2020). Speech-based characterization of dopamine replacement therapy in people with Parkinson’s disease. npj Parkinson’s Dis..

[9] Birba, A. et al. Multimodal neurocognitive markers of naturalistic discourse typify diverse neurodegenerative diseases. *Cerebral Cortex*, 10.1093/cercor/bhab421 (2021). 10.1093/cercor/bhab421 PMC9376869 34875690

[10] Dorsey ER (2018). Global, regional, and national burden of Parkinson’s disease, 1990-2016: a systematic analysis for the global burden of disease study 2016. Lancet Neurol..

[11] Postuma RB, Berg D (2016). Advances in markers of prodromal Parkinson disease. Nat. Rev. Neurol..

[12] Fernandino L (2013). Parkinson’s disease disrupts both automatic and controlled processing of action verbs. Brain Lang..

[13] Péran P (2009). Object naming and action-verb generation in Parkinson’s disease: a fMRI study. Cortex.

[14] Abrevaya S (2017). The road less traveled: alternative pathways for action-verb processing in Parkinson’s disease. J. Alzheimer’s Dis..

[15] Herrera E, Cuetos F (2012). Action naming in Parkinson’s disease patients on/off dopamine. Neurosci. Lett..

[16] Bocanegra Y (2017). Unspeakable motion: selective action-verb impairments in parkinson’s disease patients without mild cognitive impairment. Brain Lang..

[17] Aarsland, D. et al. Mild cognitive impairment in parkinson disease: a multicenter pooled analysis. *Neurology* **75**, 1062–1069 (2010). 10.1212/WNL.0b013e3181f39d0e PMC2942065 20855849

[18] Yarnall AJ (2010). (2014). Characterizing mild cognitive impairment in incident parkinson disease: the ICICLE-PD study. Neurology.

[19] Williams-Gray CH, Foltynie T, Brayne CE, Robbins TW, Barker RA (2007). Evolution of cognitive dysfunction in an incident Parkinson’s disease cohort. Brain.

[20] García AM (2016). How language flows when movements don’t: an automated analysis of spontaneous discourse in Parkinson’s disease. Brain Lang..

[21] Birba, A. et al. Motor-system dynamics during naturalistic reading of action narratives in first and second language. *NeuroImage* 116820, 10.1016/j.neuroimage.2020.116820 (2020). 10.1016/j.neuroimage.2020.116820 PMC7412856 32278096

[22] Trevisan P, García AM (2019). Systemic functional grammar as a tool for experimental stimulus design: New appliable horizons in psycholinguistics and neurolinguistics. Lang. Sci..

[23] Burciu RG, Vaillancourt DE (2018). Imaging of motor cortex physiology in Parkinson’s disease. Mov. Disord..

[24] Binder JR, Desai RH, Graves WW, Conant LL (2009). Where is the semantic system? A critical review and meta-analysis of 120 functional neuroimaging studies. Cereb. Cortex.

[25] Melzer TR (2012). Grey matter atrophy in cognitively impaired Parkinson’s disease. J. Neurol. Nurosurgery Psychiatry.

[26] Hoops S (2009). Validity of the MOCA and MMSE in the detection of MCI and dementia in Parkinson disease. Neurology.

[27] Hu M (2011). How well do we recognise non-motor symptoms in a British Parkinson’s disease population?. J. Neurol..

[28] García AM (2021). Cognitive determinants of dysarthria in Parkinson’s disease: an automated machine learning approach. Mov. Disorderds.

[29] Sanz C (2022). Automated text-level semantic markers of Alzheimer’s disease. Alzheimer’s Dement.: Diagnosis Assess. Dis. Monit..

[30] Boulenger V (2008). Word processing in Parkinson’s disease is impaired for action verbs but not for concrete nouns. Neuropsychologia.

[31] Crescentini, C., Mondolo, F., Biasutti, E. & Shallice, T. Supervisory and routine processes in noun and verb generation in nondemented patients with Parkinson’s disease. *Neuropsychologia* **46**, 434–447 (2008). 10.1016/j.neuropsychologia.2007.08.021 17931671

[32] Signorini M, Volpato C (2006). Action fluency in Parkinson’s disease: a follow-up study. Mov. Disord..

[33] Cardona JF (2014). How embodied is action language? Neurological evidence from motor diseases. Cognition.

[34] Herrera E, Cuetos F, Ribacoba R (2012). Verbal fluency in Parkinson’s disease patients on/off dopamine medication. Neuropsychologia.

[35] Eyigoz E (2020). From discourse to pathology: automatic identification of Parkinson’s disease patients via morphological measures across three languages. Cortex.

[36] García AM, Orozco-Arroyave JR (2021). Reply to: “Does cognitive impairment influence motor speech performance in de novo parkinson’s disease”. Mov. Disord..

[37] Hughes AJ, Daniel SE, Kilford L, Lees AJ (1992). Accuracy of clinical diagnosis of idiopathic parkinson’s disease: A clinico-pathological study of 100 cases. J. Neurol. Neurosurg. Psychiatry.

[38] Fahn, S. & Elton, R. L. in *Recent Developments in Parkinson’s Disease II* (eds Fahn, S., Marsden, C. D. & Goldstein, M.) 153–163 (Macmillan, 1987).

[39] Hoehn MM, Yahr MD (1967). Parkinsonism: onset, progression and mortality. Neurology.

[40] Torralva T, Roca M, Gleichgerrcht E, López P, Manes F (2009). INECO Frontal Screening (IFS): a brief, sensitive, and specific tool to assess executive functions in dementia. J. Int. Neuropsychol. Soc..

[41] Mahoney FI, Barthel DW (1965). Functional evaluation: the Barthel Index. Md. State Med. J..

[42] Lawton, M. P. & Brody, E. M. Assessment of older people: self-maintaining and instrumental activities of daily living. *Gerontologist* **9**, 179–186 (1969). 5349366

[43] Litvan I (1969). (2012). Diagnostic criteria for mild cognitive impairment in Parkinson’s disease: Movement disorder society task force guidelines. Mov. Disord..

[44] Nasreddine ZS (2005). The Montreal Cognitive Assessment, MOCA: a brief screening tool for mild cognitive impairment. J. Am. Geriatr. Soc..

[45] Pereira-Manrique F, Reyes M (2013). Confiabilidad y validez del test montreal cognitive assessment (moca) en población mayor de bogotá colombia. Rev. Neuropsicología Neuropsiquiatría Neurocienc..

[46] Novotný M, Rusz J, Čmejla R, Růžička E (2014). Automatic evaluation of articulatory disorders in parkinson’s disease. IEEE/ACM Trans. Audio Speech Lang. Process..

[47] Tomlinson CL (2010). Systematic review of levodopa dose equivalency reporting in Parkinson’s disease. Mov. Disord..

[48] Birba, A. et al. Electrifying discourse: anodal TDCS of the primary motor cortex selectively reduces action appraisal in naturalistic narratives. *Cortex*, 10.1016/j.cortex.2020.08.005 (2020). 10.1016/j.cortex.2020.08.005 PMC7655702 32950239

[49] Klumpp, P., Fritsch, J. & Noeth, E. ANN-based Alzheimer’s disease classification from bag of words, In: *Speech Communication; 13th ITG-Symposium,* pp. 1–4. https://ieeexplore.ieee.org/document/8578051 (2018).

[50] Vrana SR (2018). Latent semantic analysis: a new measure of patient-physician communication. Soc. Sci. Med..

[51] San Miguel Abella RA, González-Nosti M (2020). Motor content norms for 4,565 verbs in spanish. Behav. Res. Methods.

[52] Honnibal M, Montani I. Spacy 2: Natural language understanding with bloom embeddings, convolutional neural networks and incremental parsing (2017).

[53] Vallat R (2018). Pingouin: Statistics in python. J. Open Source Softw..

[54] Perez-Toro, P. A., Vasquez-Correa, J. C., Bocklet, T., Noth, E. & Orozco-Arroyave, J. R. User state modeling based on the arousal-valence plane: applications in customer satisfaction and health-care. *IEEE Trans. Affective Comput.* 1–1, 10.1109/TAFFC.2021.3112543 (2021).

[55] Burges CJC (1998). A tutorial on support vector machines for pattern recognition. Data Min. Knowl. Discov..

[56] Schölkopf B, Smola AJ (2002). Learning with Kernels..

[57] Bergstra J, Bengio Y (2012). Random search for hyper-parameter optimization. J. Mach. Learn. Res..

[58] Khattak FK (2019). A survey of word embeddings for clinical text. J. Biomed. Inform..

[59] Bravo-Candel D, López-Hernández J, García-Díaz JA (2021). Automatic correction of real-word errors in Spanish clinical texts. Sensors.

