# Supplementary material for: Detecting Parkinson’s disease and its cognitive phenotypes via automated semantic analyses of action stories
Source: NPJ Parkinsons Dis. 2022 Nov 25;8:163. doi: 10.1038/s41531-022-00422-8 (PMC9700793; doi:10.1038/s41531-022-00422-8)
Supplement: Supplementary file 1 — Supplementary information [file 41531_2022_422_MOESM1_ESM.pdf]

Supplementary Information

1. Demographic and clinical data for each sub-group

Table S1. Demographic and clinical data for the sub-groups of patients and controls.

	PD-nMCI patients	HCs for PD-nMCI patients	PD-nMCI patients vs. HCs	PD-MCI patients	HCs for PD-MCI patients	PD-MCI patients vs. HCs	PD-nMCI versus PD-MCI
	$n = 24$	$n = 24$	p-value	$n = 16$	$n = 16$	p-value	p-value
**Sociodemographic variables**							
Sex (F:M)	10:14	9:15	.77[a]	5:11	6:10	.71[a]	.50[a]
Age	61.13 (9.67)	60.46 (7.38)	.74[b]	63.94 (8.66)	64.00 (6.91)	.92[b]	.45[b]
Years of education	12.08 (5.04)	12.29 (4.63)	.80[b]	12.44 (5.14)	13.44 (4.69)	.66[b]	.94[b]
**Clinical variables**							
Years since diagnosis	5.30 (3.31)	-----	-----	6.16 (4.34)	-----	-----	.67[b]
UPDRS-III	27.46 (10.11)	-----	-----	36.31 (14.22)	-----	-----	.06[b]
H&Y	1.98 (.23)	-----	-----	2.16 (.35)	-----	-----	.09[b]
IFS	20.54 (2.73)	23.04 (2.99)	**< .001**[b]	17.63 (3.59)	22.13 (2.22)	**< .001**[b]	**.01**[b]
Barthel Index	100 (0)	-----	-----	100 (0)	-----	-----	1.0[b]
L&B	8 (0)	-----	-----	8 (0)	-----	-----	1.0[b]
MoCA	26.83 (1.34)	27.21 (1.41)	.39[b]	21.63 (1.78)	25.94 (1.69)	**< .001**[b]	**< .001**[b]
LED	602.21 (396.94)	-----	-----	714.34 (353.06)	-----	-----	.26[b]

Data presented as mean (*SD*); *p*-values calculated using (a) chi-squared tests and (b) Mann-Whitney *U* tests. PD: Parkinson's disease; PD-nMCI: Parkinson's disease without mild cognitive impairment; PD-MCI: Parkinson's disease with mild cognitive impairment; HCs: healthy controls; UPDRS-III: Unified Parkinson's Disease Rating Scale, part III; H&Y: Hoehn & Yahr scale; IFS: INECO Frontal Screening; L&B: Lawton & Brody Index; MoCA: Montreal Cognitive Assessment; LED: Levodopa equivalent dose.

2. Texts and approximate English translations

Action text: Spanish original

Sábado por la tarde. ¡El momento favorito de Juancito en toda la semana! Tomó a sus padres de la mano y juntos corrieron hasta la plazoleta. Al lado de las hamacas, un grupo de niños aplaudía las ocurrencias de un colorido payaso. Juancito corrió velozmente hacia el lugar donde el payaso saltaba y bailaba sin cesar. Al terminar el espectáculo, el payaso escribió su nombre en el pavimento. ¡Qué sorpresa! ¡También se llamaba Juan! Luego, entre toda la muchedumbre, Juancito caminó hacia el banco donde se sentaron sus padres. Abrazó a su padre con mucha fuerza y le jaló la camisa para que se levantara. ¡Era hora de jugar al fútbol! Juancito tomó la pelota y la puso en el césped. Su padre se movía de izquierda a derecha, en posición de arquero. Juancito pateó y… ¡gol! De repente salió el sol. Juancito se sacó el suéter y lo apoyó en el banco. Luego, su madre se acercó y le entregó un chocolate. Se lo comió de un bocado. Como siempre, al terminarlo, arrojó el envoltorio en el basurero. Había sido una intensa jornada. Se sentía muy cansado. Ya en la falda de su madre, mientras se limpiaba los restos de golosina de la boca, se quedó dormido.

Action text: Approximate English translation

Saturday afternoon. Juancito’s favorite time of the week! He grabbed his parents by the hand and ran with them to the park. Next to the swings, a group of children applauded the antics of a colorful clown. Juancito ran quickly to the place where the clown jumped and danced non-stop. When the show was over, the clown wrote his name on the pavement. What surprise! He was also called Juan! Then, among the crowd, Juancito walked to the bench where his parents were sitting. He hugged his father with strength and pulled him by the shirt to have him stand up. It was time to play soccer! Juancito grabbed the ball and put it on the grass. His father moved from left to right, pretending to be a goalkeeper. Juancito kicked the ball and… goal! Suddenly, the sun appeared. Juancito removed his sweater and placed it on the bench. Then, his mother approached him and handed him a chocolate. He ate it in a single bite. As usual, upon finishing it, he threw the wrapping in the trash can. It had been a very intense day. He felt very tired. Once on his mother’s lap, while he wiped bits of chocolate off his mouth, he fell asleep.

Non-action text: Spanish original

La noche recién comenzaba. Alberto estaba eufórico. ¡Gracias a Dios por los fines de semana! A unas pocas cuadras, la discoteca. Sus amigos lo aguardaban allí y juntos compartirían un buen momento. Al cruzar la calle, Alberto leyó el nombre de la discoteca en un cartel: “Ni jefe ni reloj”. Siempre lo ponía de buen humor. Una vez adentro, lo encandilaron las luces. Sintió calor y se encontró muy transpirado. Al lado del bar, un grupo de mujeres se entretenía con las ocurrencias de su amigo, Mario. Las muchachas se reían sin pausa junto a ese joven que bromeaba e inventaba personajes. Luego, entre toda la gente, Alberto reconoció a su novia, Elsa. Ella lo esperaba en una silla. Desde atrás, Alberto le preguntó si le gustaba la música. “¡Por supuesto!”, respondió Elsa. Aunque ella tenía sueño, Alberto le insistió para que lo acompañara a la pista. ¡Era hora de disfrutar la música! Como siempre, al decidirse, Elsa se olvidó la cartera en la silla. Alberto escuchó su canción favorita y se entusiasmó mucho. Elsa, fiel compañera, lo ayudó a recordar la letra. ¡Qué buen equipo! De regreso en su casa, mientras sentía el sudor y el cansancio en el cuerpo, se quedó dormido.

Non-action text: Approximate English translation

The night was just beginning. Alberto was euphoric. Thank God for weekends! The disco was just a few blocks away. His friends were waiting for him there to have a good a time together. Upon crossing the street, Alberto read the name of the disco on a sign: “No boss, no clock”. It always cheered him up. Once inside, he was blinded by lights. He felt the heat and found himself soaked in wet. Next to the bar, a group of women were entertained by the bantering of his friend, Mario. The girls were laughing non-stop with that young man who joked and invented characters. Then, among the crowd, Alberto recognized his girlfriend, Elsa. She was waiting for him sitting on a chair. From behind, Alberto asked her whether she liked the music. “Of course!,” replied Elsa. Though she was sleepy, Alberto insisted that she joined him on the dancing floor. It was time to enjoy the music! As usual, upon deciding, Elsa forgot her purse on the chair. Alberto heard his favorite song and was very excited. Elsa, ever the good sport, helped him remember the lyrics. What a good team! On the way back home, as he felt the sweat and tiredness all over his body, Alberto fell asleep.

References

1 Davis, C. J. & Perea, M. BuscaPalabras: a program for deriving orthographic and phonological neighborhood statistics and other psycholinguistic indices in Spanish. *Behavior research methods* **37**, 665-671 (2005).

2 Szigrist Pazos, F. *Sistemas de legibilidad del mensaje escrito: fórmula de perspicuidad* Ph.D. thesis, Universidad Complutense de Madrid, (1993).

3 Barrio-Cantalejo, I. M. *et al.* Validación de la Escala INFLESZ para evaluar la legibilidad de los textos dirigidos a pacientes. *Anales del Sistema Sanitario de Navarra* **31**, 135-152 (2008).
