# Supplementary material for: Detecting Parkinson’s disease and its cognitive phenotypes via automated semantic analyses of action stories
Source: NPJ Parkinsons Dis. 2022 Nov 25;8:163. doi: 10.1038/s41531-022-00422-8 (PMC9700793; doi:10.1038/s41531-022-00422-8)
Supplement: Supplementary file 2 — Reporting Summary [file 41531_2022_422_MOESM2_ESM.pdf]

nature portfolio

Corresponding author(s): Adolfo M. García, Juan Rafael Orozco-Arroyave | NPJPARKD-01197

Last updated by author(s): 2022/10/21

Reporting Summary

Nature Portfolio wishes to improve the reproducibility of the work that we publish. This form provides structure for consistency and transparency in reporting. For further information on Nature Portfolio policies, see our Editorial Policies and the Editorial Policy Checklist.

Statistics

For all statistical analyses, confirm that the following items are present in the figure legend, table legend, main text, or Methods section.

n/a	Confirmed	
☐	☒	The exact sample size (*n*) for each experimental group/condition, given as a discrete number and unit of measurement
☐	☒	A statement on whether measurements were taken from distinct samples or whether the same sample was measured repeatedly
☐	☒	The statistical test(s) used AND whether they are one- or two-sided<br>*Only common tests should be described solely by name; describe more complex techniques in the Methods section.*
☐	☒	A description of all covariates tested
☐	☒	A description of any assumptions or corrections, such as tests of normality and adjustment for multiple comparisons
☐	☒	A full description of the statistical parameters including central tendency (e.g. means) or other basic estimates (e.g. regression coefficient) AND variation (e.g. standard deviation) or associated estimates of uncertainty (e.g. confidence intervals)
☐	☒	For null hypothesis testing, the test statistic (e.g. *F*, *t*, *r*) with confidence intervals, effect sizes, degrees of freedom and *P* value noted<br>*Give P values as exact values whenever suitable.*
☐	☒	For Bayesian analysis, information on the choice of priors and Markov chain Monte Carlo settings
☐	☒	For hierarchical and complex designs, identification of the appropriate level for tests and full reporting of outcomes
☐	☒	Estimates of effect sizes (e.g. Cohen's *d*, Pearson's *r*), indicating how they were calculated

Our web collection on statistics for biologists contains articles on many of the points above.

Software and code

Policy information about availability of computer code

Data collection	Audio recordings were obtained via WaveSurfer 1.8.8p4.
Data analysis	Statistical analyses were performed on Pingouin, an open-source statistical package.

For manuscripts utilizing custom algorithms or software that are central to the research but not yet described in published literature, software must be made available to editors and reviewers. We strongly encourage code deposition in a community repository (e.g. GitHub). See the Nature Portfolio guidelines for submitting code & software for further information.

Data

Policy information about availability of data

All manuscripts must include a data availability statement. This statement should provide the following information, where applicable:

- Accession codes, unique identifiers, or web links for publicly available datasets
- A description of any restrictions on data availability
- For clinical datasets or third party data, please ensure that the statement adheres to our policy

The datasets generated and/or analyzed during the current study are available in the Open Science Framework (OSF) repository under the title "García (2022). Semantics of retelling in PD", https://osf.io/6xc5b/, doi: 10.17605/OSF.IO/6XC5B

Human research participants

Policy information about studies involving human research participants and Sex and Gender in Research.

Reporting on sex and gender	The term 'sex' has been used to indicate a biological attribute. Findings apply to both sexes, as noted in the manuscript. Sex was determined via self-reports, as noted in the manuscript. Disaggregated sex data has been provided in the source data. Our sample includes 15 women and 25 men with Parkinson's disease, as well as 15 women and 25 men in healthy condition (as reported in Table 5). No separate analyses were performed on each sex given that no specific hypotheses were raised for this variable in the study design.
Population characteristics	As detailed in Table 5, patients had a mean of 62.25 years of age, 12.23 years of education, 5.65 years since diagnosis, 31.0 UPDRS-III score, 2.05 H&Y score, 19.56 IFS battery score, 100 Barthel Index score, 8 Lawton & Brody score, 24.75 MoCA score, and 658.27 Levodopa equivalent dose. Healthy controls had a mean of 61-87 years of age, 12.75 years of education, 22.92 IFS battery score, and 26.7 MoCA score.
Recruitment	Participants were recruited from an ongoing protocol at a movement disorders clinic in Medellín, Colombia, by postings and talks organized to invite participants.
Ethics oversight	The study was approved by the Institutional Ethics Committee of Antioquia University (resolutions 14-10-569 and 15-10-569).

Note that full information on the approval of the study protocol must also be provided in the manuscript.

Field-specific reporting

Please select the one below that is the best fit for your research. If you are not sure, read the appropriate sections before making your selection.

☐ Life sciences ☒ Behavioural & social sciences ☐ Ecological, evolutionary & environmental sciences

For a reference copy of the document with all sections, see nature.com/documents/nr-reporting-summary-flat.pdf

Behavioural & social sciences study design

All studies must disclose on these points even when the disclosure is negative.

Study description	The study is quantitative experimental.
Research sample	Patients with Parkinson's disease and healthy controls. Patients had a mean of 62.25 years of age, 12.23 years of education, 5.65 years since diagnosis, 31.0 UPDRS-III score, 2.05 H&Y score, 19.56 IFS battery score, 100 Barthel Index score, 8 Lawton & Brody score, 24.75 MoCA score, and 658.27 Levodopa equivalent dose. Healthy controls had a mean of 61-87 years of age, 12.75 years of education, 22.92 IFS battery score, and 26.7 MoCA score. The study was chosen given its compliance with inclusion/exclusion criteria and accessibility for testing.
Sampling strategy	A convenience sampling strategy was employed. The sample size surpasses the mean of previous linguistic studies on Parkinson's disease, and it allowed for cross-validation analyses, guaranteeing generalizability of results. Detection of robust results in previous studies with similar or smaller sample sizes pointed to the adequacy of the present sample size.
Data collection	A professional microphone and audio software were used for speech data acquisition. Neuropsychological and demographic data were obtained with pen and paper tests. Nobody was present during testing other than the participants and the examiner. The examiner was blind to the study hypotheses during data collection.
Timing	Start: 2015/11/11 | Finish: 2016/01/18
Data exclusions	No data were excluded from the analyses.
Non-participation	No participants dropped out or declined participation
Randomization	Participants were allocated into the Parkinson's disease or healthy control groups based on their health status.

Reporting for specific materials, systems and methods

We require information from authors about some types of materials, experimental systems and methods used in many studies. Here, indicate whether each material, system or method listed is relevant to your study. If you are not sure if a list item applies to your research, read the appropriate section before selecting a response.

Materials & experimental systems

n/a	Involved in the study
☐	☐ Antibodies
☐	☐ Eukaryotic cell lines
☐	☐ Palaeontology and archaeology
☐	☐ Animals and other organisms
☐	☒ Clinical data
☐	☐ Dual use research of concern

Methods

n/a	Involved in the study
☐	☐ ChIP-seq
☐	☐ Flow cytometry
☐	☐ MRI-based neuroimaging

Antibodies

Antibodies used	No antibodies were used.
Validation	Not applicable.

Eukaryotic cell lines

Policy information about cell lines and Sex and Gender in Research

Cell line source(s)	Not applicable.
Authentication	Not applicable.
Mycoplasma contamination	Not applicable.
Commonly misidentified lines (See ICLAC register)	Not applicable.

Palaeontology and Archaeology

Specimen provenance	Not applicable.
Specimen deposition	Not applicable.
Dating methods	Not applicable.

☐ Tick this box to confirm that the raw and calibrated dates are available in the paper or in Supplementary Information.

Ethics oversight	Not applicable.

Note that full information on the approval of the study protocol must also be provided in the manuscript.

Animals and other research organisms

Policy information about studies involving animals; ARRIVE guidelines recommended for reporting animal research, and Sex and Gender in Research

Laboratory animals	Not applicable.
Wild animals	Not applicable.
Reporting on sex	Not applicable.
Field-collected samples	Not applicable.
Ethics oversight	Not applicable.

Note that full information on the approval of the study protocol must also be provided in the manuscript.

Clinical data

Policy information about clinical studies
All manuscripts should comply with the ICMJE guidelines for publication of clinical research and a completed CONSORT checklist must be included with all submissions.

Clinical trial registration	Not applicable.
Study protocol	Not applicable.
Data collection	Not applicable.
Outcomes	Not applicable.

Dual use research of concern

Policy information about dual use research of concern

Hazards

Could the accidental, deliberate or reckless misuse of agents or technologies generated in the work, or the application of information presented in the manuscript, pose a threat to:

No	Yes	
☒	☐	Public health
☒	☐	National security
☒	☐	Crops and/or livestock
☒	☐	Ecosystems
☒	☐	Any other significant area

Experiments of concern

Does the work involve any of these experiments of concern:

No	Yes	
☒	☐	Demonstrate how to render a vaccine ineffective
☒	☐	Confer resistance to therapeutically useful antibiotics or antiviral agents
☒	☐	Enhance the virulence of a pathogen or render a nonpathogen virulent
☒	☐	Increase transmissibility of a pathogen
☒	☐	Alter the host range of a pathogen
☒	☐	Enable evasion of diagnostic/detection modalities
☒	☐	Enable the weaponization of a biological agent or toxin
☒	☐	Any other potentially harmful combination of experiments and agents

ChIP-seq

Data deposition

☐ Confirm that both raw and final processed data have been deposited in a public database such as GEO.

☐ Confirm that you have deposited or provided access to graph files (e.g. BED files) for the called peaks.

Data access links<br>*May remain private before publication.*	Not applicable.
Files in database submission	Not applicable.
Genome browser session<br>(e.g. UCSC)	Not applicable.

Methodology

Replicates	Not applicable.
Sequencing depth	Not applicable.

Antibodies	Not applicable.
Peak calling parameters	Not applicable.
Data quality	Not applicable.
Software	Not applicable.

Flow Cytometry

Plots

Confirm that:

- [ ] The axis labels state the marker and fluorochrome used (e.g. CD4-FITC).
- [ ] The axis scales are clearly visible. Include numbers along axes only for bottom left plot of group (a 'group' is an analysis of identical markers).
- [ ] All plots are contour plots with outliers or pseudocolor plots.
- [ ] A numerical value for number of cells or percentage (with statistics) is provided.

Methodology

Sample preparation	Not applicable.
Instrument	Not applicable.
Software	Not applicable.
Cell population abundance	Not applicable.
Gating strategy	Not applicable.

- [ ] Tick this box to confirm that a figure exemplifying the gating strategy is provided in the Supplementary Information.

Magnetic resonance imaging

Experimental design

Design type	Not applicable.
Design specifications	Not applicable.
Behavioral performance measures	Not applicable.

Acquisition

Imaging type(s)	Not applicable.
Field strength	Not applicable.
Sequence & imaging parameters	Not applicable.
Area of acquisition	Not applicable.

Diffusion MRI [ ] Used [x] Not used

Preprocessing

Preprocessing software	Not applicable.
Normalization	Not applicable.
Normalization template	Not applicable.
Noise and artifact removal	Not applicable.
Volume censoring	Not applicable.

Statistical modeling & inference

Model type and settings	Not applicable.
Effect(s) tested	Not applicable.

Specify type of analysis: ☐ Whole brain ☐ ROI-based ☐ Both

Statistic type for inference (See Eklund et al. 2016)	Not applicable.
Correction	Not applicable.

Models & analysis

n/a	Involved in the study
☒	☐ Functional and/or effective connectivity
☒	☐ Graph analysis
☒	☐ Multivariate modeling or predictive analysis
